# Heme oxygenase-1 promotes neuron survival through down-regulation of neuronal NLRP1 expression after spinal cord injury

**DOI:** 10.1186/s12974-016-0521-y

**Published:** 2016-02-29

**Authors:** Wen-Ping Lin, Gong-Peng Xiong, Qing Lin, Xuan-Wei Chen, Li-Qun Zhang, Jin-Xing Shi, Qing-Feng Ke, Jian-Hua Lin

**Affiliations:** Department of Orthopedic Surgery, the Second Affiliated Hospital, Fujian Medical University, Quanzhou, 362000 China; Hepatology Unit, Xiamen Hospital of Traditional Chinese Medicine, Xiamen, 361009 China; Department of Human Anatomy, Histology and Embryology, School of Basic Medical Sciences, Fujian Medical University, Fuzhou, 350108 China; Department of Orthopedic Surgery, the First Affiliated Hospital, Fujian Medical University, Fuzhou, 350004 China

## Abstract

**Background:**

Understanding the mechanisms underlying neuronal death in spinal cord injury (SCI) and developing novel therapeutic approaches for SCI-induced damage are critical for functional recovery. Here we investigated the role of heme oxygenase-1 (HO-1) in neuroprotection after SCI.

**Methods:**

Adeno-associated virus expressing HO-1 was prepared and injected into rat spinal cords before SCI model was performed. HO-1 expression, inflammasome activation, and the presence of inflammatory cytokines were determined by quantitative polymerase chain reaction, immunohistological staining, immunoblot, and immunoprecipitation. Neuronal apoptosis was assessed by terminal deoxynucleotidyl transferase dUTP nick end labeling. The hindlimb locomotor function was evaluated for extent of neurologic damage. In an in vitro model, hydrogen peroxide was used to induce similar inflammasome activation in cultured primary spinal cord neurons, followed by evaluation of above parameters with or without transduction of HO-1-expressing adeno-associated virus.

**Results:**

Endogenous HO-1 expression was found in spinal cord neurons after SCI in vivo, in association with the expression of Nod-like receptor protein 1 (NLRP1) and the formation of NLRP1 inflammasomes. Administration of HO-1-expressing adeno-associated virus effectively decreased expression of NLRP1, therefore alleviating NLRP1 inflammasome-induced neuronal death and improving functional recovery. In the in vitro model, exogenous HO-1 expression protected neurons from hydrogen peroxide-induced neuronal death by inhibiting NLRP1 expression. In addition, HO-1 inhibited expression of activating transcription factor 4 (ATF4), which is a transcription factor regulating NLRP1 expression.

**Conclusions:**

HO-1 protects spinal cord neurons after SCI through inhibiting NLRP1 inflammasome formation.

**Electronic supplementary material:**

The online version of this article (doi:10.1186/s12974-016-0521-y) contains supplementary material, which is available to authorized users.

## Background

Spinal cord injury (SCI) leads to complex cellular and molecular interactions within the central nervous system (CNS) in an attempt to repair the initial tissue damage. The pathophysiology of SCI is characterized by the shearing of cell membranes and axons, disruption of the blood–spinal cord barrier, cell death, immune cell transmigration, and myelin degradation [1]. There are two mechanisms of damage to the spinal cord after injury: the primary mechanical injury and the secondary injury mediated by multiple injury processes [2]. SCI-induced neuronal death in the lesion area seems to be the consequence of both the primary injury and the secondary injury depending on its localization and temporal process [3, 4]. Several molecular biological processes, including changes of cell cycle-related gene expression, endoplasmic reticulum (ER) stress, glutamate excitotoxicity, free radical production, and inflammatory cytokine release contribute to neuronal death [1, 5–8]. Recently, inflammasome-associated neuronal programmed cell death, termed pyroptosis, has been shown to contribute to neuronal death in distinct neurological diseases [9–12]. Pyroptosis is induced by inflammasomes which consist of an apoptosis-associated speck-like protein containing a caspase recruitment domain (ASC), an adaptor protein, and caspase-1, an inflammatory cysteine–aspartic protease [13, 14]. The formation of inflammasomes activates caspase-1 and subsequently leads to plasma-membrane pore formation and cleavage of chromosomal DNA. Caspase-1 dependence is a defining feature of pyroptosis, and caspase-1 is the enzyme that mediates this process of cell death [15]. In addition, caspase-1, also known as interleukin-1-converting enzyme, plays an important role in the inflammatory processes by cleaving pro-IL-1β into mature pro-inflammatory IL-1β [16]. IL-1β can be produced and released by CNS neurons following distinct stimulation and insults, suggesting that neurons are also a source of neuroinflammation [9, 17–20]. Inflammasome activation and formation have been shown to be present in the CNS cells including spinal cord neurons after CNS injury. For example, CNS trauma promotes the expression of the NOD-like receptor protein-1 (NLRP1), ASC, and caspase-1 in spinal cord motor neurons and cortical neurons [21]. NLRP1 inflammasome formation occurs in neurons after stroke in rodents [19]. NLRP1 inflammasomes are activated in patients with medial temporal lobe epilepsy and contribute to neuronal pyroptosis in the amygdala kindling-induced rat model [12]. Therefore, inhibition of inflammasome-mediated neuronal death could be neuroprotective in several neurological disorders.

Heme oxygenases (HO) are evolutionarily conserved enzymes that catabolize heme into equimolar amounts of labile Fe, carbon monoxide, and biliverdin [22]. HO have two types: HO-1 expression is induced ubiquitously in response to various stresses, whereas HO-2 is constitutively expressed and can be further induced by opioids and glucocorticoids [23–27]. HO and its by-product carbon monoxide were found to blunt the stress axis activation secondary to many stimuli including immune-inflammatory stressors [28–30]. A previous study showed that both HO-1 messenger RNA (mRNA) and protein are elevated in the ventral horn motor neurons shortly after experimental SCI [31]. In addition, HO-1 modulates the secondary injury process, and high HO-1 expression may preserve spinal cord function in the early stages after SCI [32]. Moreover, HO-1 stabilizes the blood–spinal cord barrier and limits oxidative stress and white matter damage in acutely injured murine spinal cord [33]. Therefore, induction of HO-1 expression could be a therapeutic approach for SCI. However, the relationship between HO-1 and inflammasome-mediated neuronal death has not been studied in SCI.

In this study, we examined the neuroprotective role of HO-1 in SCI and explored the feasibility of using HO-1-expressing adeno-associated virus (AAV) as a therapeutic approach for alleviating neuronal death in the acute phase of SCI. We found that NLRP1 inflammasomes were assembled and activated in neurons in the injury region after SCI. HO-1 expression was also induced in neurons at the same region shortly after SCI. In an in vitro model, we found that exogenous HO-1 expression protected neurons from NLRP1 inflammasome-mediated neuronal death by inhibiting NLRP1 expression, conversely silencing endogenous HO-1 expression with small interfering RNA (siRNA) increased neuronal death. In addition, HO-1 inhibited expression of activating transcription factor 4 (ATF4), which is a transcription factor regulating NLRP1 expression. Moreover, administration of HO-1-expressing AAV into the spinal cord before SCI effectively decreased the expression of ATF4 and NLRP1 after SCI, therefore alleviating neuronal death and improving functional recovery. Taken together, our study indicated that HO-1 protects spinal cord neurons through inhibition of NLRP1 inflammasome formation.

## Methods

### Animals and SCI model

Adult male Sprague–Dawley rats (280–320 g) were used in this study. The animal experiments were performed in accordance with the guidelines of Laboratory Animals and were approved by the Animal Care and Use Committee of Fujian Medical University. Spinal cord compression at T12 was performed following an established static compression model [34]. Briefly, animals were anesthetized by inhalation of 2–3 % isoflurane administered at a flow rate of 1 L/min. Midline skin incisions were performed to expose the T12 spinous processes. A laminectomy was performed at T12. The compression was applied by suspending the base of a compression platform (area 2 × 5 mm^2^) onto the exposed cord. A weight of 50 g was applied statically to the platform for exactly 5 min. After removing the platform, the muscles and skins were sutured. In the sham group, only a laminectomy was performed.

### Primary spinal cord neuron culture and H_2_O_2_ treatment

Primary spinal cord neuron culture was conducted following the established method with minor modifications [35, 36]. Briefly, spinal cords were dissected from E16 rat embryos. Isolated spinal cords free of meninges and dorsal root ganglia were digested with 0.25 % Trypsin in Hank’s balanced salt solution (HBSS, Hyclone) at 37 °C for 30 min. The cell suspension was titrated in Dulbecco’s Modified Eagle Medium (DMEM) supplemented with 1 % penicillin–streptomycin and 10 % fetal bovine serum (FBS, Hyclone). Then 8 × 10^5^ cells, 4 × 10^5^ cells, or 1 × 10^5^ cells were seeded into each well of poly-l-lysine-coated 6-well plates, 24-well plates, or 96-well plates, respectively. To treat primary neurons, H_2_O_2_ (Sigma-Aldrich) was added into the neuron culture at the final concentration of 10 μM.

### Preparation of adeno-associated virus

To produce AAV expressing HO-1 (termed A-HO-1 thereafter), HO-1 complementary DNA (cDNA) was amplified by PCR and inserted to *BamHI* sites of pAAV-CMV-EGFP and confirmed by sequencing. The recombinant plasmid was then packaged into AAV2/8 particles (AAV2 ITRs; AAV8 capsid). All these procedures were conducted by Neuronbiotech Company. The A-HO-1 titer was 5.5 × 10^12^ genome copies/ml, and the titer of EGFP-expression control AAV2/8 virus (termed A-C hereafter) was 7.5 × 10^12^ genome copies/ml. Viral titer was determined with quantitative PCR by the detection of WPRE element copies of AAV genome.

### AAV administration in vitro and in vivo

To transduce primary neurons, on day 7 in vitro, 2.0 × 10^11^ genome copies of AAV in 50 μl of PBS were added into each well of six-well plates containing 1.5 × 10^5^ cells in 1.5 ml medium and were incubated for 24 h before further processing or treatment.

To assess the effect of A-HO-1 in vivo, the rats were randomly assigned to three groups including vehicle, A-C and the A-HO-1 groups. Seven days before SCI, 3 μl of normal saline was injected into the spinal cord of the vehicle group rats at T12 at a rate of 0.2 μl/min using a 5-μl micro-syringe with a 33-gauge needle (Hamilton). At the same time point using the same method, 3 μl of A-C or A-HO-1 was injected into the rats of A-C group or A-HO-1 group, respectively. After injection, the injectors were removed and the muscles and skins were sutured in separate layers.

### siRNA transfection

siRNAs for HO-1, NLRP1, and ATF4 were purchased from Santa Cruz Biotechnolgy. siRNAs (50 pmol each) were transfected into 1.5 × 10^5^ primary neurons using RNAiMAX reagent (Life Technologies) according to manufacturer’s protocol. After 24 h, cells were subject to further processing including immunoblot and H_2_O_2_ treatment.

### Immunofluorescent staining

The spinal cord was fixed with 4 % paraformaldehyde and then was embedded in paraffin using a Leica APS300. Five-micron cross sections were prepared, and antigen retrieval was performed by boiling sections in sodium citrate buffer (10 mM sodium citrate, 0.05 % Tween 20, pH 6.0) for 30 min. Anti-NLRP1 (Abcam), anti-HO-1 (Santa Cruz Biotechnology), and anti-MAP2 (Millipore) antibody were used for staining according to the manufacturer’s instructions. Alexa Fluor 488 donkey anti-rabbit IgG and Alexa Fluor 594 chicken anti-goat IgG (both from Invitrogen) were used as secondary antibodies. The sections were observed on a Leica DMIRE2 inverted fluorescent microscope.

### Terminal deoxynucleotidyl transferase dUTP nick end labeling (TUNEL)

Primary neuron culture or spinal cord sections were labeled with DeadEnd™ Fluorometric TUNEL System (Promega) following the manufacturer’s manual. The sections were observed on a Leica DMIRE2 inverted fluorescent microscope.

### RNA isolation, reverse transcription, and quantitative RT-PCR (q-RTPCR)

Total RNA was extracted from cells or tissues using the RNeasy Mini Kit (Qiagen). cDNA synthesis was performed using SuperScript^®^ III First-Strand Synthesis System (Invitrogen). q-RTPCR was performed using Fast SYBR^®^ Green Master Mix (Invitrogen) on a 7300 q-RTPCR System (Invitrogen). Data was analyzed with 7300 system software (Invitrogen). Primer sequences for each gene are as follows: β-actin (5′-ACAACCTTCTTGCAGCTCCTC-3′ and 5′-CTGACCCATACCCACCATCAC-3′). TNF-α (5′-TCGGTCCCAACAAGGAGGAG-3′) and (5′-GGGCTTGTCACTCGAGTTTTG-3′). IL-1β (5′-TGTCTGACCCATGTGAGCTG-3′) and (5′-GCCACAGGGATTTTGTCGTT-3′). NLRP1 (5′-GTGGCTGGACCTCTGTTTGA-3′) and (5′-GGCGTTTCTAGGACCATCCC-3′). NLRP3 (5′-CCAGAGCCTCACTGAACTGG-3′) and (5′-AGCATTGATGGGTCAGTCCG-3′). NLRC4 (5′-AGGCAAACTGGATTTGCTTGG-3′) and (5′-TGTGGTGAGTCAAACCGTCC-3′). ASC (5′-GGACAGTACCAGGCAGTTCG-3′) and (5′-GTCACCAAGTAGGGCTGTGT3′). Caspase-1 (5′-GACCGAGTGGTTCCCTCAAG-3′) and (5′-GACGTGTACGAGTGGGTGTT-3′). ATF4 (5′-ACCATGGCGTATTAGAGGCA-3′) and (5′-GCTGGTATCGAGGAATGTGC-3′). PCR conditions used for all primer sets were as follows: 95 °C hot start for 10 min, followed by 40 amplification cycles of 95 °C for 15 s (denaturing), 60 °C for 1 min (annealing, extension, and detection). Relative abundance of RNA was analyzed using 2^−ΔΔCt^ method.

### Immunoblot assay

The following antibodies were used for immunoblot: anti-β-actin, anti-ASC, anti-HO-1, anti-HO-2, anti-ATF4, and anti-CHOP antibodies were purchased from Cell Signaling Technology. Anti-NLRP1, anti-NLRP3, anti-NLRC4, anti-AIM2, and anti-pro-caspase-1 antibodies were purchased from Abcam. Anti-caspase-1 antibody (against both pro-caspase-1 and p20) was purchased from Santa Cruz Biotechnology. Anti-IL-1β (against both pro-IL-1β and mature IL-1β) was purchased from Novus Biologicals.

### Co-immunoprecipitation

To assess the formation of NLRP1 inflammasome, spinal cord tissues or cultured neurons were lysed in 50 mM Tris-HCl (pH 7.4), 150 mM NaCl, 0.5 % NP-40, and complete protease inhibitors (Roche). Five hundred micrograms of spinal cord lysates from sham or SCI animals, or primary neuron homogenates, were incubated on ice for 15 min and then centrifuged at 2600×*g* for 5 min at 4 °C to remove tissue/cell debris. Immunoprecipitation assays were performed using 1 mg magnetic Dynabeads (Invitrogen) coupled to 7 mg of anti-NLRP1 (Abcam) or anti-ASC (Cell Signaling Technology) antibody according to the manufacturer’s instructions. Briefly, 250 μg of lysate applied to 1.5 mg of anti-NLRP1 or anti-ASC antibody-coupled magnetic beads, incubated overnight at 4 °C on a rotator, and eluted in a low pH buffer. Pre-immune serum (Abnova) served as a negative control. Elutions were collected and added to ×1 Laemmli buffer to dissociate proteins. Five microliters of 1 M Tris (pH 9.8) were added to match the pH of the stacking gel. Samples were then boiled for 5 min and subjected to SDS-PAGE and immunoblot analysis with indicated antibodies.

### Neurologic evaluation

The hindlimb locomotor function was assessed at pre-injury and 1, 3, 7, 14, and 21 days after SCI using the BBB locomotor test developed by Basso et al. [37]. The hindlimb movements during locomotion were quantified using a scale ranging from 0 to 21. The rats were observed for 5 min at each time point by two observers who were blinded to the experimental protocol.

### Statistics

Data was presented as mean ± SD and analyzed by statistical software (Prism 6.0, GraphPad Software). Student’s *t* test or one-way ANOVA was used for comparison of mean between the groups. For BBB score analysis, repeated-measures ANOVA was used. *p* values <0.05 were considered significant.

## Results

### SCI induces expression of inflammasome components

**Fig. 1:**
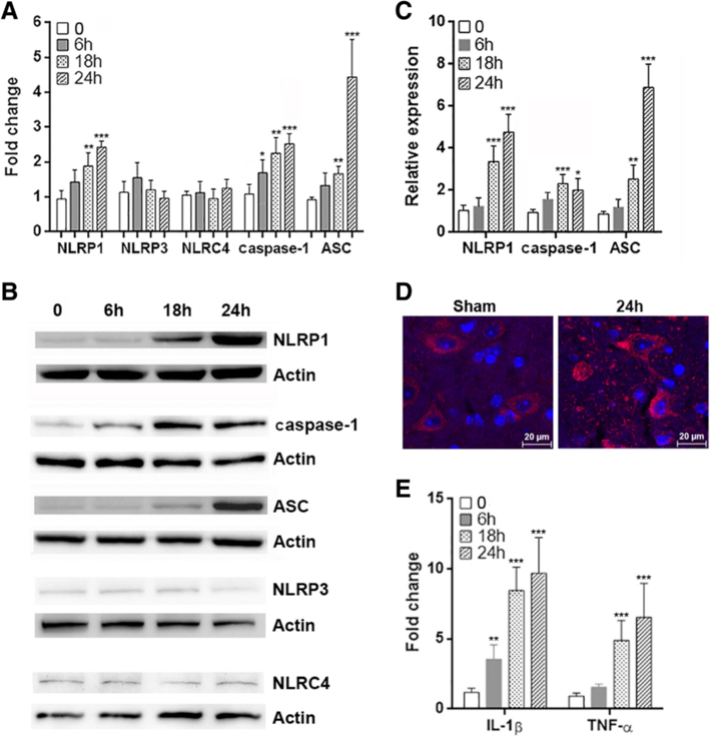
SCI induces expression of inflammasome components in the spinal cord. **a** mRNA levels of inflammasome components in injured spinal cords were determined by q-RTPCR at different time points after SCI. **b** Protein levels of inflammasome components in injured spinal cords were determined by immunoblot at different time points after SCI. **c** Statistical analysis for protein levels of inflammasome components in injured spinal cords after SCI. **d** Immunofluorescent staining of NLRP1 in spinal cords. **e** mRNA levels of IL-1β and TNF-α in the injured spinal cords were determined by q-RTPCR at different time points after SCI. 0: immediately after SCI. *N* = 6 per group. *p < 0.05; **p < 0.01; ***p < 0.001 in comparison with the “0” group

The activation of inflammasome pathways in neurological disorders including SCI has been documented previously [38]. To investigate the temporal dynamics and types of inflammasomes after SCI, we firstly determined mRNA levels of different inflammasome components in the injury region at different time points after SCI. We found that NLRP1 mRNA was significantly elevated from 18 to 24 h after SCI. NLRP3 mRNA was slightly up-regulated at 6 h but returned to basal level at 18 h. There was not significant change of NLRC4 mRNA level. Caspase-1 mRNA and ASC mRNA were also up-regulated after SCI (Fig. 1a). Immunoblot showed consistent increases in the protein levels of NLRP1, caspase-1, and ASC (Fig. 1b, c). However, protein levels of NLRP3 and NLRC4 were not significantly altered (Fig. 1b). The increase of NLRP1 expression was confirmed by histological staining, and it seemed that neurons express NLRP1 (Fig. 1d). The expression of pro-inflammatory cytokines IL-1β and TNF-α was also increased at 6 h after SCI and remained at high levels until 24 h (Fig. 1e).

### SCI induces NLRP1 inflammasome activation

**Fig. 2:**
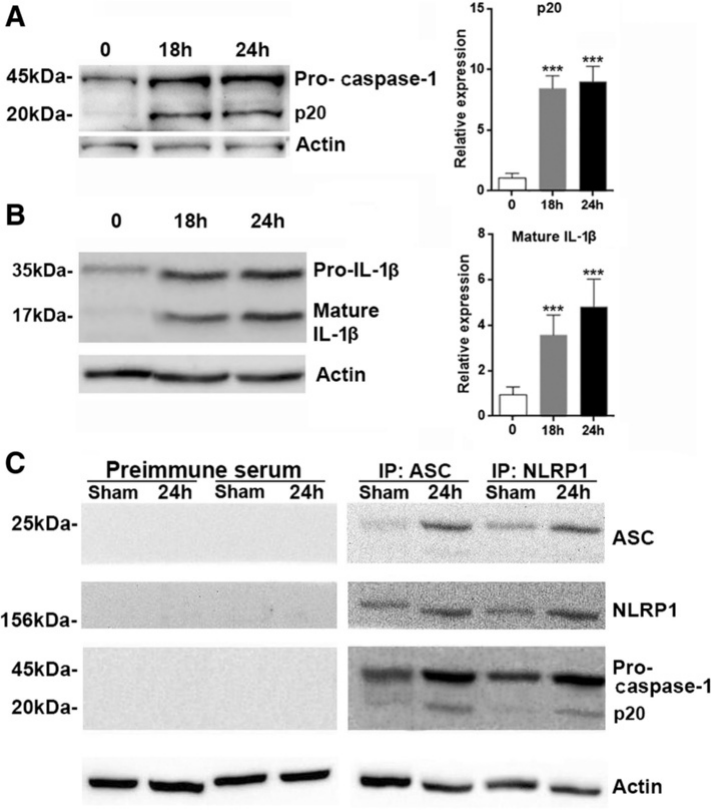
NLRP1 inflammasomes are formed in the spinal cord after SCI. **a** Expression of pro-caspase-1 and caspase-1 p20 in injured spinal cords after SCI. *Left panel*: representative immunoblot image. *Right panel*: statistical analysis for caspase-1 p20. 0: immediately after SCI. **b** Expression of pro-IL-1β and mature IL-1β in injured spinal cords after SCI. *Left panel*: representative immunoblot image. *Right panel*: statistical analysis for mature IL-1β. 0: immediately after SCI. **c** Association of ASC, NLRP1, and caspase-1 in injured spinal cords was determined by co-immunoprecipitation. *N* = 6 per group. ***p < 0.001 in comparison with the “0” group

To determine whether NLRP1 inflammasomes are formed after SCI, the processing of pro-caspase-1 and pro-IL-1β was tested. SCI induced significant increases in both pro-caspase-1 and caspase-1 p20 levels, suggesting that pro-caspase-1 was substantially cleaved to become activated caspase-1 by inflammasome formation (Fig. 2a). Consistently, mature IL-1β level was also up-regulated after SCI, suggesting that activated caspase-1 cleaved pro-IL-1β into mature IL-1β (Fig. 2b). To characterize the association of inflammasome proteins after SCI, co-immunoprecipitations of post-SCI spinal cord lysates were performed using anti-ASC antibody, anti-NLRP1 antibody, and pre-immune serum, respectively. As shown in Fig. 2c, in the sham spinal cord, ASC was immunoprecipitated with anti-ASC antibody, and low levels of NLRP1 and caspase-1 were present in this signaling complex. At 24 h after SCI, there was increased association of NLRP1 and caspase-1with ASC. In addition, the pro-caspase-1 associated to ASC was cleaved to generate activated caspase-1, demonstrated by increased immunoprecipitated caspase-1 p20. Consistently, immunoprecipitation with anti-NLRP1 antibody showed increased association of ASC and pro-caspase-1 with NLRP1, in comparison with the sham control. As a negative control, pre-immune serum did not immunoprecipitate the inflammasome-associated proteins, indicating the antibody specificity. Taken together, our data suggested that SCI not only induced expression of NLRP1 inflammasome components but also triggered the assembly of inflammasomes.

### SCI induces HO-1 expression in spinal cord neurons

**Fig. 3:**
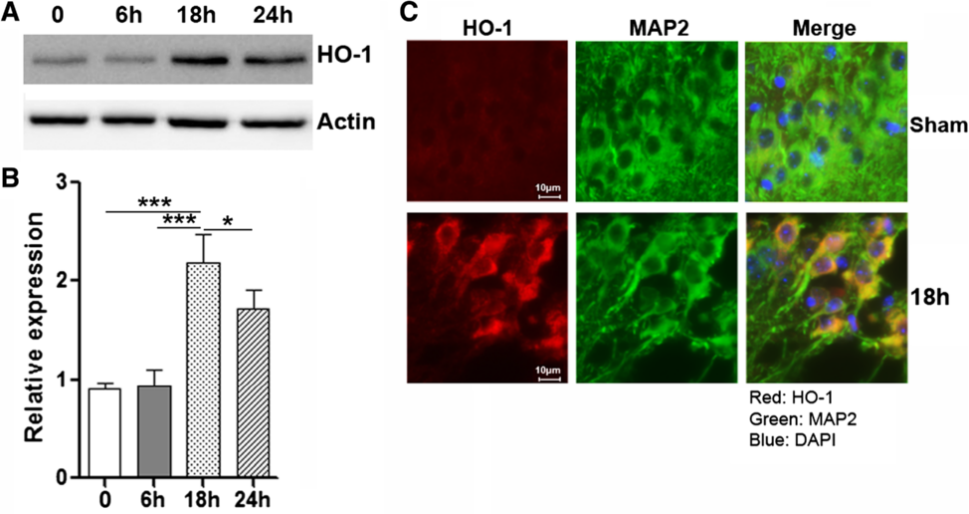
SCI induces HO-1 expression in the spinal cord. **a** HO-1 expression in injured spinal cords was determined by immunoblot at different time points after SCI. 0: immediately after SCI. **b** Statistical analysis for HO-1 protein level in injured spinal cords after SCI. 0: immediately after SCI. **c** HO-1 expression in MAP2^+^ spinal cord neurons was indicated by immunofluorescent staining. *N* = 6 per group. *p < 0.05; ***p < 0.001 in comparison with sham control

HO-1 has been shown to be induced after SCI [31]. To confirm this, we examined expression of HO-1 in the SCI region. As shown in Fig. 3a, b, HO-1 protein level was relatively low immediately after SCI and was not changed at 6 h after SCI. However, at 18 h after SCI, its expression was remarkably up-regulated and stayed high until 24 h. Immunofluorescent staining revealed enhanced HO-1 expression in MAP2^+^ neurons, suggesting that SCI induced HO-1 expression in spinal cord neurons (Fig. 3c).

### HO-1 inhibits NLRP1 expression to alleviate neuronal death induced by hydrogen peroxide

**Fig. 4:**
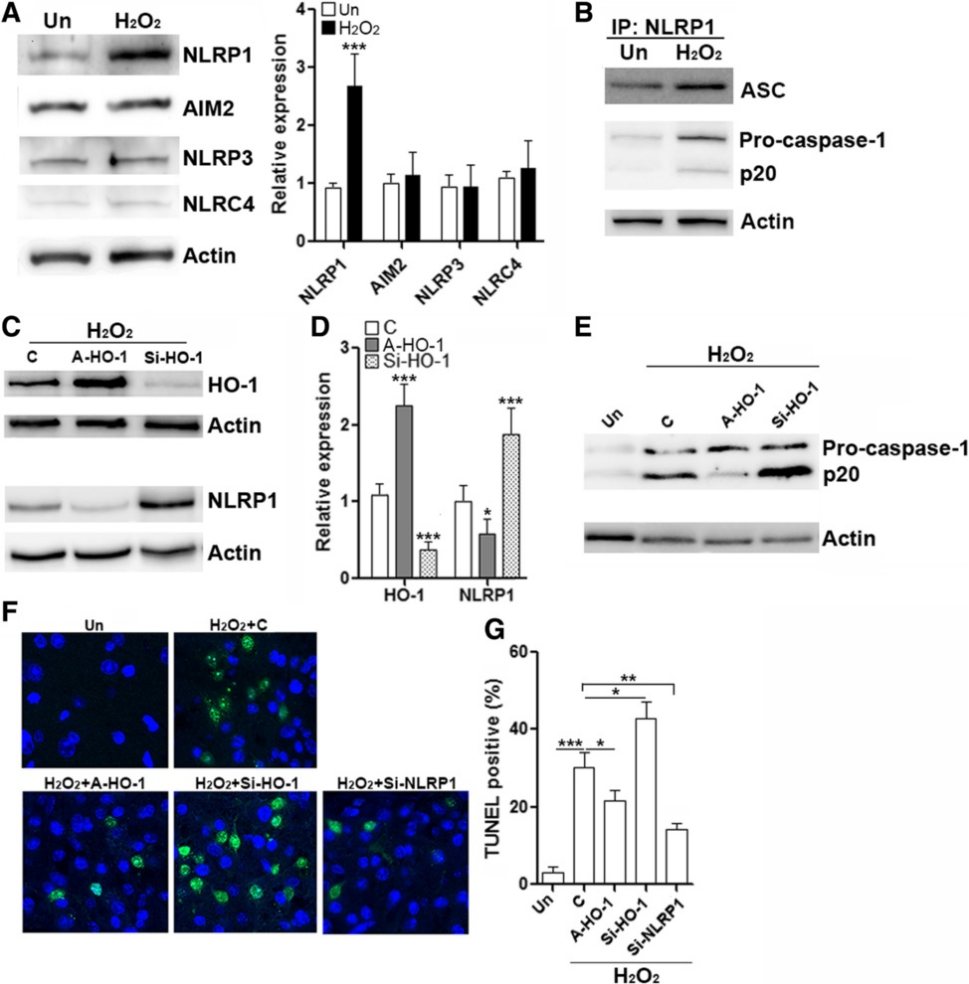
HO-1 protects spinal cord neurons from NLRP1 inflammasome-induced neuronal death. **a** H_2_O_2_ induced NLRP1 expression in primary spinal cord neurons after 6-h treatment. *Left panel*: representative immunoblot image. *Right panel*: statistical analysis for inflammasome components. *Un*: untreated control. **b** Association of NLRP1 with ASC and caspase-1 was determined by co-immunoprecipitation after 6-h H_2_O_2_ treatment. *Un*: untreated control. **c** HO-1 inhibited NLRP1 expression after H_2_O_2_ treatment. Primary neurons were transduced with A-HO-1 or transfected with HO-1 siRNA. Twenty four hours later, neurons were treated with H_2_O_2_ for additional 6 h. Expression of HO-1 and NLRP1 was determined by immunoblot. This is a representative image of four independent experiments. *C*: cells under transduction or transfection condition without AAV and siRNA. *Si-HO-1*: HO-1 siRNA. **d** Statistical analysis for the expression of HO-1 and NLRP1 in **c**. *p < 0.05; ***p < 0.001 compared with “C” group. **e** Expression of pro-caspase-1 and caspase-1 p20 in H_2_O_2_-treated primary neurons. *Un*: untreated control. *C*: cells under transduction or transfection condition without AAV and siRNA. *Si-HO-1*: HO-1 siRNA. **f** Representative images of TUNEL staining after 12-h H_2_O_2_ treatment. *Un*: untreated control. *C*: cells under transduction or transfection condition without AAV and siRNA. *Si-HO-1*: HO-1 siRNA. *Si-NLRP1*: NLRP1 siRNA. **g** Statistical analysis for TUNEL. *N* = 6 per group. *p < 0.05; **p < 0.01; ***p < 0.001

To investigate the effect of HO-1 on neuronal survival, we employed an in vitro model using H_2_O_2_ to induce neuronal damage. Primary spinal neurons were treated with H_2_O_2_ for 6 h, and inflammasome signaling was evaluated. Similar to SCI, H_2_O_2_ induced a significant increase in NLRP1 expression compared with untreated control, while expression of AIM2, NLRP3, and NLRC4 was not changed (Fig. 4a). Co-immunoprecipitation indicated that the association of ASC and pro-caspase-1 with NLRP1 was weak in untreated neurons, whereas H_2_O_2_ promoted the association of NLRP1 with ASC and pro-caspase-1, suggesting enhanced formation of NLRP1 inflammasomes (Fig. 4b). Of note, in H_2_O_2_-treated neurons, NLRP1-associated caspase-1 p20 was also higher than that in untreated neurons, suggesting caspase-1 activation in H_2_O_2_-treated neurons (Fig. 4b). Therefore, H_2_O_2_ triggered a pattern of inflammasome activation similar to SCI and presented a viable in vitro model to simulate SCI-induced inflammasome activation. We then manipulated the expression of HO-1 in this model by transfection of HO-1 siRNA or transduction of HO-1-expressing AAV (A-HO-1) into primary spinal neurons. In our preliminary study, HO-1 siRNA inhibited HO-1 expression while not altering HO-2 (Additional file 1: Figure S1A). Transduction of control AAV (A-C) did not significantly change the expression of HO-1 and NLRP1 (Additional file 1: Figure S1B), suggesting that AAV transduction procedure cannot alter HO-1 and NLRP1 expression. In addition, neither transfection of scramble siRNA nor transduction of control AAV triggered caspase-1 activation, suggesting that inflammasome formation was not induced by the transfection or transduction procedures themselves (Additional file 1: Figure S1C). After H_2_O_2_ treatment, compared with vehicle control, A-HO-1 significantly increased HO-1 expression while down-regulating NLRP1 expression (Fig. 4c, d). Conversely, HO-1 siRNA reduced HO-1 expression while up-regulating NLRP1 level (Fig. 4c, d). This data suggested that HO-1 negatively regulated NLRP1 expression in H_2_O_2_-treated neurons. Consistent with the change of NLRP1 expression, A-HO-1 significantly reduced caspase-1 activation, demonstrated by lower level of caspase-1 p20 when compared to vehicle control (Fig. 4e). HO-1 siRNA remarkably increased caspase-1 p20 level (Fig. 4e), suggesting that HO-1 inhibited NLRP1 expression and subsequently decreased NLRP1 inflammasome-induced caspase-1 activation.

To investigate whether NLRP1 expression was related to neuronal death, we transfected primary neurons with NLRP1 siRNA before H_2_O_2_ treatment. NLRP1 siRNA effectively decreased NLRP1 expression and did not influence the expression of NLRP3 and NLRC4 (Additional file 1: Figure S1D and S1E). Neuronal death was evaluated by TUNEL at 12 h after H_2_O_2_ treatment. We found that H_2_O_2_ induced substantial TUNEL-positive cells, while A-HO-1 decreased TUNEL-positive cells, and HO-1 siRNA robustly increased TUNEL-positive cells (Fig. 4f, g). NLRP1 siRNA also significantly reduced TUNEL-positive cells after H_2_O_2_ treatment (Fig. 4f, g). Scramble siRNA transfection and control AAV transduction did not significantly altered TUNEL staining (Additional file 1: Figure S2). Thus, our data indicated that NLRP1 induced neuronal death and HO-1 inhibited neuronal death through inhibiting NLRP1 expression.

### HO-1 regulates NLRP1 expression through ATF4

**Fig. 5:**
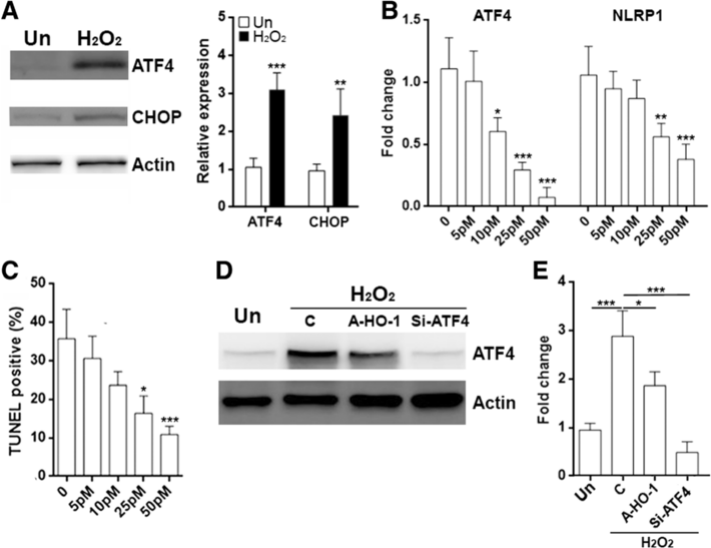
HO-1 inhibits NLRP1 expression through ATF4. **a** Expression of ATF4 and CHOP in primary neurons were determined by immunoblot after 6-h H_2_O_2_ treatment. *Left panel*: representative immunoblot image. *Right panel*: statistical analysis for the expression of ATF4 and CHOP. *Un*: untreated control. *N* = 4 per group. ***p < 0.001 compared with “Un” group. **b** mRNA levels of ATF4 and NLRP1 in primary neurons. Primary neurons were transfected with different doses of ATF4 siRNA 48 h before treatment with H_2_O_2_ for additional 6 h. *N* = 3 per group. *p < 0.05; **p < 0.01; ***p < 0.001 compared with “0” group. **c** Neurons were treated as in **b**, followed by detection of neuronal death with TUNEL after 12-h H_2_O_2_ treatment. *N* = 6 per group. *p < 0.05; ***p < 0.001 compared with “0” group. **d** Protein level of ATF4 in primary neurons. Primary neurons were transduced with A-HO-1 or transfected with ATF4 siRNA for 24 h before treatment with H_2_O_2_ for additional 6 h. *Un*: untreated control. *C*: cells under transduction or transfection condition without AAV and siRNA. *Si-ATF4*: siRNA for ATF4. This is a representative of three independent experiments. **e** Neurons were treated as in **d**. mRNA level of ATF4 was determined by q-RTPCR. *N* = 5 per group. **p < 0.01; ***p < 0.001

A recent study indicates that transcription factor ATF4 induces NLRP1 expression during ER stress [39]. In addition, ER stress contributes to cell death and survival in a SCI model [40]. Therefore, we tested whether HO-1 could regulate NLRP1 expression via ATF4. We firstly checked the expression of ATF4 and its target C/EBP homology protein (CHOP) in H_2_O_2_-treated primary spinal cord neurons. H_2_O_2_ significantly up-regulated ATF4 and CHOP levels (Fig. 5a). To evaluate the role of ATF4 in regulating NLRP1 expression, we transfected primary spinal cord neurons with different concentrations of ATF4 siRNA before H_2_O_2_ treatment. As shown in Fig. 5b, the ATF4 mRNA level was decreased by ATF4 siRNA in a dose-dependent manner. Of note, the NLRP1 mRNA level was also decreased in association with the decrease of ATF4 mRNA, suggesting that ATF4 positively regulated NLRP1 expression. To investigate the role of ATF4 in H_2_O_2_-induced neuronal death, TUNEL was performed. We found that ATF4 siRNA inhibited neuronal death in a dose-dependent manner (Fig. 5c). We then evaluated the effect of exogenous HO-1 on ATF4 expression by transducing neurons with A-HO-1. When compared to non-transduced control cells, A-HO-1 significantly reduced ATF4 protein level (Fig. 5d) and mRNA level (Fig. 5e). To exclude the possibility that AAV transduction itself influences ATF4 expression, A-C was transduced into both untreated and H_2_O_2_-treated neurons. We found insignificant changes in ATF4 mRNA level between transduced and non-transduced neurons, suggesting AAV itself did not alter ATF4 expression (Additional file 1: Figure S3). Therefore, the change in A-HO-1-transfected neurons should be ascribed to the effect of HO-1.

### HO-1 protects neurons from SCI in vivo

**Fig. 6:**
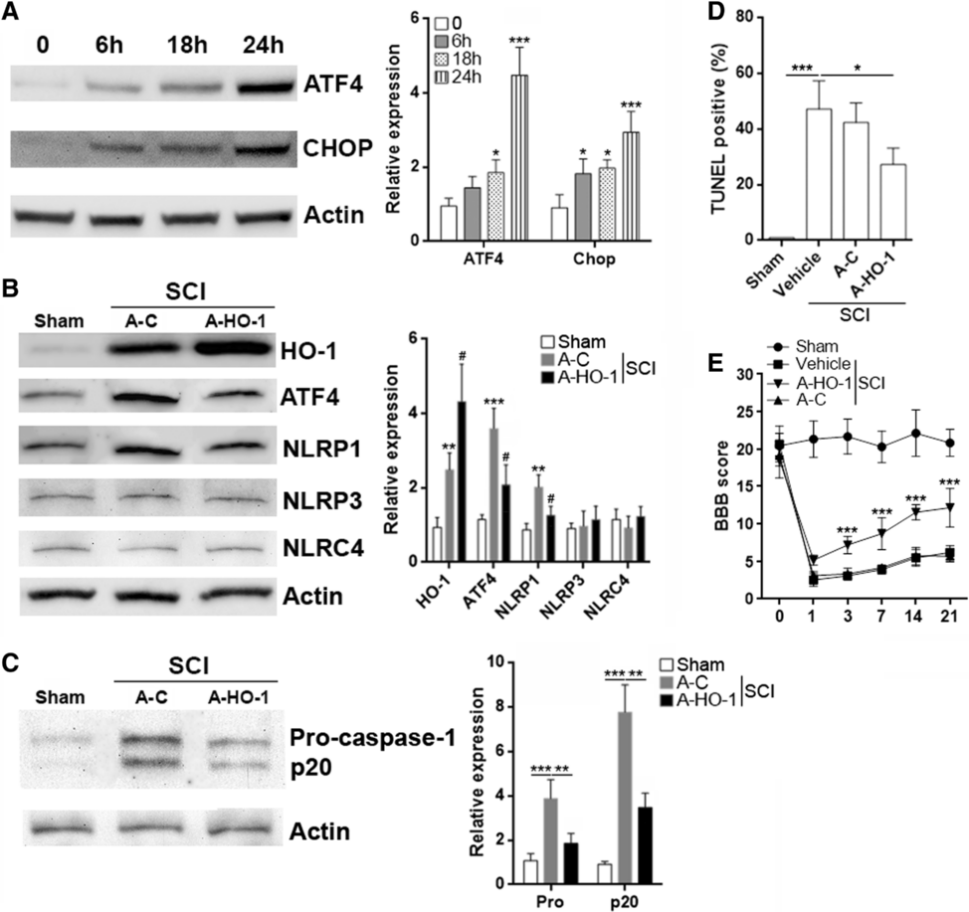
Administration of A-HO-1 protects neurons from SCI-induced neuronal death in vivo. **a** Expression of ATF4 and CHOP in injured spinal cords after SCI. *Left panel*: representative immunoblot image. *Right panel*: statistical analysis for the expression of ATF4 and CHOP. 0: immediately after SCI. *N* = 3 per group. *p < 0.05; ***p < 0.001 compared with “0” group. **b** Expression of HO-1, ATF4, and inflammasome components in injured spinal cords 24 h after SCI. *Left panel*: representative immunoblot image. *Right panel*: statistical analysis for the expression of each protein. *Sham*: sham control. *N* = 5 per group. **p < 0.01; ***p < 0.001 compared with sham control. ^#^ *p* < 0.05 compared with A-C group. **c** Expression of pro-caspase-1 and caspase-1 p20 in injured spinal cords 24 h after SCI. *Left panel*: representative immunoblot image. *Right panel*: statistical analysis for the expression of each protein. *Sham*: sham control. *N* = 5 per group. **d** Neuronal death was quantified by TUNEL 24 h after SCI. **e** BBB score after SCI. *N* = 10 per group. *p < 0.05; **p < 0.01; ***p < 0.001

Similar to in vitro H_2_O_2_ treatment, SCI induced up-regulation of ATF4 and CHOP in the injury region of spinal cord (Fig. 6a). To evaluate whether HO-1 is neuroprotective after SCI, we injected A-C or A-HO-1 into the spinal cord 7 days before SCI. Injected A-C induced expression of EGFP (Additional file 1: Figure S4A) but did not influence the expression of ATF4, NLRP1, NLRP3, and NLRC4 in the spinal cord (Additional file 1: Figure S4B), suggesting that AAV injection successfully introduced exogenous genes into the spinal cord while not altering endogenous inflammasome-related gene expression. A-C also did not significantly alter HO-1 expression after SCI (Additional file 1: Figure S4C). A-HO-1 significantly increased HO-1 level in the post-SCI spinal cord in comparison with A-C (Fig. 6b). In addition, A-HO-1 significantly reduced ATF4 and NLRP1 expression after SCI compared with A-C but did not change the expression of NLRP3 and NLRC4 (Fig. 6b). Consistent with lower NLRP1 level, the expression of caspase-1 p20 was down-regulated by A-HO-1 (Fig. 6c). Interestingly, the expression of pro-caspase-1 was also reduced by A-HO-1 (Fig. 6c), suggesting that HO-1 might directly or indirectly inhibited caspase-1 expression in vivo. TUNEL revealed that A-HO-1 effectively decreased neuronal death after SCI (Fig. 6d). To assess the change of hindlimb locomotor function after SCI, the BBB score at pre-injury and 1, 3, 7, 14, and 21 days after SCI are shown in Fig. 6e. All rats initially showed a slight decrease in the BBB score, and there was no significant difference among the four groups before SCI. The sham group showed full recovery 3 days after SCI. The vehicle, A-C and A-HO-1 groups showed a sharp decrease in the BBB score 1 day after SCI. Partial improvements were observed, and there was no significant difference among the vehicle and A-C group after SCI. However, the neurological improvements were significantly greater in the A-HO-1 group compared to the improvements in the vehicle and A-C groups at 3, 7, 14, and 21 days after SCI.

## Discussion

In this study, we examined the role of HO-1 in regulating NLRP1 inflammasome formation and neuronal death after SCI. SCI consists of a primary mechanical injury and a secondary inflammatory injury and apoptosis [1]. The primary injury is locally restricted to the area of the vertebral fracture and is characterized by acute hemorrhage and ischemia. The secondary injury is characterized by further destruction of neurons and glial cells and leads to significant expansion of the damage, so that paralysis can extend to higher segments. After SCI, cell death at the lesion site comprises both post-traumatic necrosis and apoptosis [41]. A time course analysis reveals that apoptosis occurs as early as 4 h post injury and can be seen in decreasing amounts as late as 3 weeks after SCI [42]. Apoptosis was observed in both neurons and oligodendrocytes. Activation of caspase-3 or 9 occurs in neurons at the injury site within hours and in neurons distant from the injury site over a period of days. Although activation of caspase-3 or 9 contributes to neuronal death, neuronal death can also be induced by caspase-1, which is activated directly via a CARD-containing inflammasome. Therefore, successful assembly and activation of inflammasomes are critical for the initiation of neuronal death. Although inflammasomes are generally formed in inflammatory immune cells, accumulating evidences have indicated that distinct types of inflammasomes are also present in neurons. NLRP1 inflammasomes mediate neuron injury under high glucose [43]. High extracellular potassium opens pannexin channels leading to inflammasome activation in primary neurons and astrocytes [44]. Importantly, recent studies have showed inflammasome formation in different neuronal disorders including SCI [12, 21, 38, 45].

Our study confirmed NLRP1 inflammasome formation in the spinal cord neurons after SCI. Interestingly, among the three NOD-like receptor proteins examined, only NLRP1 level was elevated after SCI. However, another study reported NLRP3 inflammasome formation in a SCI model [46]. The main difference between our model and their’s is the spinal segmental levels receiving SCI: ours was spinal T12 and theirs was spinal T9. This disparity raised an interesting question of whether SCI performed on different spinal levels leads to activation of different inflammasome types. However, to our knowledge, no previous or current studies indicated different responses to insults/stimuli in different spinal segmental neurons. Hence, further investigation will be needed to elucidate the mechanism underlying this disparity.

In addition to elevated expression of NLRP1, ASC, and caspase-1, the association of these proteins was also up-regulated to form NLRP1 inflammasomes in injured spinal cord. The molecular signaling which triggers inflammasome assembly after SCI has been previously studied. It has been proposed that danger signals such as high plasma glucose, β-amyloid, Toll-like receptor (TLR) ligands, uric acid, and ATP induce inflammasome activation [47–51]. In particular, the engagement of ATP with P2X4 or P2X7 purinergic receptors induces inflammasome activation. Spinal cord neurons express P2X7 purinergic receptors, and exposure to ATP led to high-frequency spiking, irreversible increases in cytosolic calcium, and cell death [52]. Systemic administration of an antagonist of P2X7 receptors improves recovery after SCI [53]. P2X4 receptors are expressed in spinal cord neurons and are responsible for inflammasome activation after SCI [45]. Importantly, SCI induces increase of ATP release in peritraumatic areas [52]. Therefore, it is highly possible that excessive ATP release activates these purinergic receptors to induce NLRP1 inflammasome formation. Another possibility is that TLRs might induce post-SCI NLRP1 inflammasome formation. Although TLRs are generally considered to be expressed in microglia and astrocytes, recent research has showed that TLR expression in neurons is pivotal under both physiological and pathological conditions [54–57]. Perhaps SCI changes expression of certain TLRs which recognize danger signals after injury and subsequently causes inflammasome formation. Further study will be performed to test this hypothesis.

The induction of HO-1 after SCI is consistent with a previous report [31]. However, the exact mechanisms underlying this induction have not been completely understood. Hemin could be one of the candidates which induce HO-1 expression, since a study stated that hemin induces HO-1 in spinal cord vasculature after SCI [58]. It is possible that hemin is released from red blood cells into the interstitial space as a result of hemorrhage after SCI. Cultured cerebellar granular neurons express heme carrier protein 1 (HCP1), and HCP1 contributes to the time- and concentration-dependent accumulation of hemin in neurons, which causes substantial neurotoxicity [59]. However, to our knowledge, there is no information about HCP1 expression in spinal cord neurons. Thus, further study will be needed to investigate the effect of hemin on spinal cord neurons. Other factors, such as hypoxia, reactive oxygen species, and pro-inflammatory cytokines can also regulate HO-1 expression in different cell types [60]. Perhaps the post-SCI HO-1 expression is a combined effect of above factors rather than the effect of a single factor. Of note, the HO-1 induction peaked at 18 h after SCI and was decreased at 24 h with respect to 18 h. This dynamic change of HO-1 expression might be explained by the transcriptional repression through Bach1. Bach1 is a transcriptional repressor of heme oxygenase-1 gene. A similar decrease of HO-1 expression by Bach1 is observed in rat fibroblasts and human lung cancer cells exposed to cigarette smoke condensate in a previous study [61]. In addition, genetic ablation of Bach1 leads to up-regulation of HO-1 in myocardial ischemia/reperfusion and SCI in mice [62, 63]. Moreover, HO-1 induction is also associated with down-regulation of Bach1 in ischemic brains [64]. However, the kinetics of Bach1 expression after compressive SCI is not fully understood. Interestingly, hypoxia-inducible factor-1 (HIF-1), which is up-regulated in injured spinal cords [65], induces the expression of Bach1 [66, 67]. Therefore, it is possible that down-regulation of HO-1 at 24 h is due to up-regulation of Bach1 by HIF-1. Future investigation will be needed to verify this hypothesis.

The anti-inflammatory role of HO-1 has been reported previously. Hemin-induced HO-1 inhibits NLRP3 inflammasome formation in acute lung injury [68]. Induction of HO-1 attenuates lipopolysaccharide-induced inflammasome formation in human gingival epithelial cells [69]. However, up-regulation of HO-1 is not always anti-inflammatory. The HO activity may exert a different effect on inflammation. Navarra et al. reported that HO activation blunts the systemic response to stressors [70]. In addition, Barone et al. found that HO-1 demonstrates increased serine residue phosphorylation and oxidative post-translational modifications in hippocampus and cerebellum in Alzheimer’s disease, suggesting HO-1 as a target of oxidative damage even in the cerebellum [71]. This serine residue phosphorylation may influence the HO-1 activity [72]. Therefore, elucidation of the exact role of HO in the neuropathy including SCI needs more elaborated research designs. In our study, HO-1 inhibits post-SCI NLRP1 expression via the transcription factor ATF4, indicating HO-1 is likely anti-inflammatory in the local SCI area. To our knowledge, we are the first to report the role of HO-1 in regulating ATF4 expression. Previous studies imply that ATF4 positively regulates HO-1 expression [73, 74], but no evidence has shown the effect of HO-1 on ATF4 expression. The mechanism by which HO-1 down-regulates ATF4 is still unknown. Based on the fact that cytosolic HO-1 is able to translocate into the nuclei of certain tumor cells [75–77], it is possible that HO-1 could directly act as a transcription suppressor or indirectly regulate some transcription factors to inhibit ATF4 expression. It has been reported that HO-1 modulates subcellular distribution and activation of Nrf2 [75], suggesting HO-1 is able to influence the activity of certain transcription factors. However, in our study, HO-1 seems to be located only in the cytosol, excluding its effect in the nucleus. We speculated that HO-1 might regulate ATF4 expression through other mechanisms rather than acting as a transcription suppressor. Our ongoing study is analyzing the downstream molecules of HO-1 signaling in an effort to assess which factor is responsible for this effect.

Inflammasome-associated neuronal death has been a research interest both in basic and clinical studies. With inflammasomes, pro-caspase-1 is activated. Its function includes conversion of the pro-IL-1β and pro-IL-18 into active IL-1β and IL-18. Cell membrane integrity is destroyed as a result of micropore formation caused by caspase-1, IL-1β, and IL-18. These micropores lead to a series of processes such as cytoplasm release, cell osmotic lysis, and inflammatory reaction. In addition, Caspase-1 is involved in degradation of DNA and chromosomes because a specific endonuclease is activated by caspase-1. Caspase-1 also triggers degradation of cytoskeletal proteins. In our study, NLRP1 inflammasomes were closely related to post-SCI neuronal death. However, it is still unclear which cell death type—apoptosis, pyroptosis, or necrosis—is dominant in the acute phase of SCI. It is plausible that each death type contributes to neuronal death at a certain time period during and after SCI, depending on the duration and severity of SCI. At the beginning of SCI, the injury stress might directly cause apoptosis or necrosis when inflammasomes have not been activated. Later on, when less damaged neurons and neurons in the penumbra are under inflammatory stress, the pyroptosis could couple with apoptosis to enhance the secondary injury. A recent study showed that AIM2 and NLRP3 inflammasomes activate both apoptotic and pyroptotic death pathways [78], making the relationship between apoptosis and pyroptosis more complicated. An elaborated study on the post-SCI cell death types will be needed to elucidate the neuronal death processes during and after SCI.

Consistent with the anti-inflammatory effect of HO-1 in most cases [22, 79, 80], our study found that HO-1 inhibits inflammasome-induced neuronal death. Whether HO-1 has the similar or different efficacy in other neuronal disorders such as ischemic stroke, Alzheimer’s disease, and multiple sclerosis needs further investigation. Our study demonstrates a new mechanism by which HO-1 protects neurons from SCI and might provide new therapeutic clues for treating SCI.

## Conclusions

NLRP1 inflammasomes were assembled and activated in neurons after spinal cord injury (SCI). HO-1 expression was induced in spinal cord neurons in association with the formation of NLRP1 inflammasomes. Administration of HO-1-expressing adeno-associated virus into spinal cords before SCI effectively decreased SCI-induced NLRP1 inflammasome formation, alleviated neuronal death, and improved functional recovery. HO-1 down-regulates NLRP1 expression through inhibiting expression of ATF4. Our study demonstrates a new mechanism by which HO-1 protects neurons from SCI.

## Additional file

Additional file 1:
**Figures S1–S4.** Figure S1. Manipulation of expression of HO-1 and NLRP1. Figure S2. TUNEL after transfection with scramble siRNA or control AAV. Figure S3. ATF4 expression after transduction with control AAV. Figure S4. Administration of AAV in vivo. (DOCX 2178 kb)

## Abbreviations

AAV: adeno-associated virus
ASC: apoptosis-associated speck-like protein containing a caspase recruitment domain
ATF4: activating transcription factor 4
CNS: central nervous system
DMEM: Dulbecco’s Modified Eagle Medium
ER: endoplasmic reticulum
FBS: fetal bovine serum
HBSS: Hank’s balanced salt solution
HCP1: heme carrier protein 1
HO: heme oxygenase
IL-1β: interleukin-1β
NLRP1: tyrosine hydroxylase; nucleotide-binding-and-oligomerization domain-like receptor protein-1
SCI: spinal cord injury
TNF-α: tumor necrosis factor-α
TUNEL: terminal deoxynucleotidyl transferase dUTP nick end labeling
