# Supplementary material for: Heme oxygenase-1 promotes neuron survival through down-regulation of neuronal NLRP1 expression after spinal cord injury
Source: J Neuroinflammation. 2016 Feb 29;13:52. doi: 10.1186/s12974-016-0521-y (PMC4772494; doi:10.1186/s12974-016-0521-y)
Supplement: Additional file 1: — Figures S1–S4. Figure S1. Manipulation of expression of HO-1 and NLRP1. Figure S2. TUNEL after transfection with scramble siRNA or control AAV. Figure S3. ATF4 expression after transduction with control AAV. Figure S4. Administration of AAV in vivo. (DOCX 2178 kb) [file 12974_2016_521_MOESM1_ESM.docx]

**Supplemental information**


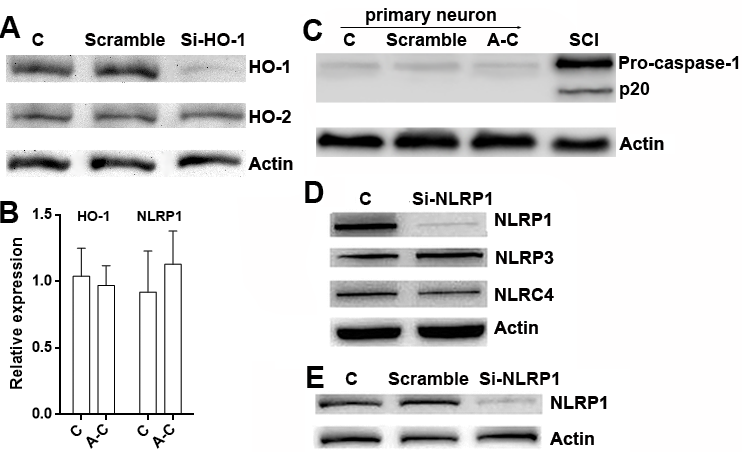


**Supplemental figure 1. Manipulation of expression of HO-1 and NLRP1.** **(A)** Inhibition of HO-1 expression by HO-1 siRNA. Primary spinal cord neurons were transfected with scramble siRNA or HO-1 siRNA for 24 h. Then neurons were treated with H_2_O_2_ for 18 h. Expression of HO-1 and HO-2 were determined by Immunoblot. This is a representative image of two independent experiments. C: vehicle control. Scramble: scramble siRNA. Si-HO-1: HO-1 siRNA. **(B)** mRNA levels of HO-1 and NLRP1 after transduction with control AAV. Primary spinal cord neurons were transduced with control AAV for 24 h. Then neurons were treated with H_2_O_2_ for additional 18 h. mRNA levels were quantitated by q-RTPCR. C: vehicle control. A-C: control AAV. N=3 per group. **(C)** Caspase-1 activation after transfection of scramble siRNA or transduction of control AAV. C: vehicle control. Scramble: scramble siRNA. A-C: control AAV. SCI: post-SCI spinal cord tissue as positive control. This is a representative image of two independent experiments. **(D)** Inhibition of NLRP1 expression by NLRP1 siRNA. Primary spinal cord neurons were transfected with NLRP1 siRNA for 24 h. Then neurons were treated with H_2_O_2_ for 18 h. Expression of NLRP1, NLRP3 and NLRC4 were determined by Immunoblot. This is a representative image of two independent experiments. C: vehicle control. Si-NLRP1: NLRP1 siRNA. **(E)** Inhibition of NLRP1 expression by NLRP1 siRNA. Primary spinal cord neurons were transfected with scramble siRNA or NLRP1 siRNA for 24 h. Then neurons were treated with H_2_O_2_ for 18 h. Expression of NLRP1 was determined by Immunoblot. This is a representative image of two independent experiments.


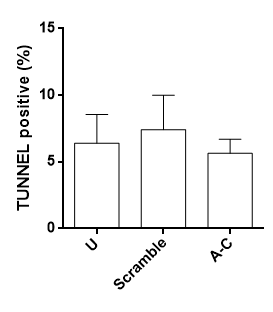


**Supplemental figure 2. TUNEL after transfection with scramble siRNA or control AAV.** Primary spinal cord neurons were transfected with scramble siRNA or transduced with control AAV for 48 h. Neuronal death was quantified by TUNEL. U: untreated control. Scramble: scramble siRNA. A-C: control AAV. N=3 per group.

**
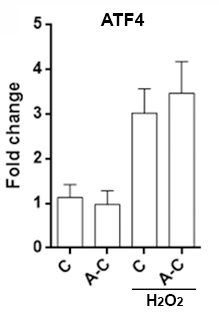
**

**Supplemental figure 3. ATF4 expression after transduction with control AAV.** Primary spinal cord neurons were transduced with control AAV for 24 h. Then neurons were treated with or without H_2_O_2_ for 18 h. Expression of AT4 was determined by qRT-PCR. C: vehicle control. A-C: control AAV. N=3 per group.


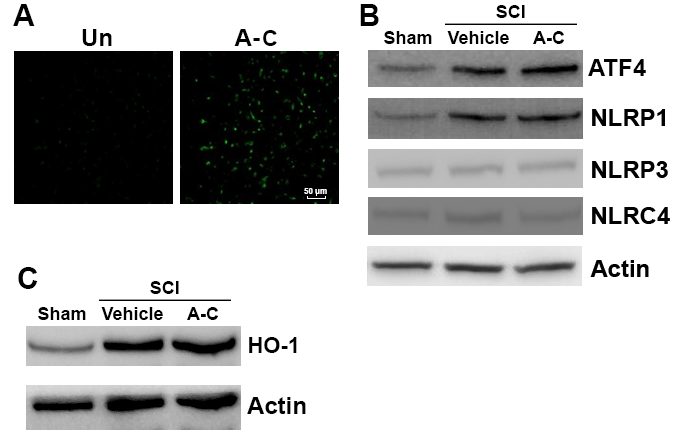


**Supplemental figure 4. Administration of AAV in vivo. (A)** *In vivo* transduction efficiency of AAV. Control AAV was injected into the spinal cord and GFP was observed in the spinal cord section. Un: untreated control. A-C: control AAV. **(B)** Control AAV was injected into the spinal cord. Then SCI was conducted. Expression of ATF4, NLRP1, NLRP3 and NLRC4 were determined by Immunoblot 24 h after SCI. Vehicle: injection of PBS. A-C: control AAV. **(C)** Control AAV was injected into the spinal cord. Then SCI was conducted. Expression of HO-1 was determined by Immunoblot 24 h after SCI. Vehicle: injection of PBS. A-C: control AAV.
